# Younger children experience lower levels of language competence and academic progress in the first year of school: evidence from a population study

**DOI:** 10.1111/jcpp.12431

**Published:** 2015-06-04

**Authors:** Courtenay Frazier Norbury, Debbie Gooch, Gillian Baird, Tony Charman, Emily Simonoff, Andrew Pickles

**Affiliations:** ^1^ Department of PsychologyRoyal HollowayUniversity of LondonEghamUK; ^2^ Newcomen CentreSt Thomas’ HospitalLondonUK; ^3^ Institute of Psychiatry, Psychology & NeuroscienceKing's College LondonLondonUK

**Keywords:** Relative age, language impairment, behaviour problems, academic achievement

## Abstract

**Background:**

The youngest children in an academic year are reported to be educationally disadvantaged and overrepresented in referrals to clinical services. In this study we investigate for the first time whether these disadvantages are indicative of a mismatch between language competence at school entry and the academic demands of the classroom.

**Methods:**

We recruited a population sample of 7,267 children aged 4 years 9 months to 5 years 10 months attending state‐maintained reception classrooms in Surrey, England. Teacher ratings on the Children's Communication Checklist‐Short (CCC‐S), a measure of language competence, the Strengths and Difficulties Questionnaire‐Total Difficulties Score (SDQ), a measure of behavioural problems, and the Early Years Foundation Stage Profile (EYFSP), a measure of academic attainment, were obtained at the end of the reception year.

**Results:**

The youngest children were rated by teachers as having more language deficits, behaviour problems, and poorer academic progress at the end of the school year. Language deficits were highly associated with behaviour problems; adjusted odds ratio 8.70, 95% CI [7.25–10.45]. Only 4.8% of children with teacher‐rated language deficits and 1.3% of those with co‐occurring language and behaviour difficulties obtained a ‘Good Level of Development’ on the EYFSP. While age predicted unique variance in academic attainment (1%), language competence was the largest associate of academic achievement (19%).

**Conclusion:**

The youngest children starting school have relatively immature language and behaviour skills and many are not yet ready to meet the academic and social demands of the classroom. At a population level, developing oral language skills and/or ensuring academic targets reflect developmental capacity could substantially reduce the numbers of children requiring specialist clinical services in later years.

## Introduction

Being among the youngest in a school year increases risk for educational and psychosocial disadvantage, increasing referrals to specialist clinical services. The youngest children in a school year experience lower levels of scholastic achievement (Cotzias & Whitehorn, 2013; Crawford, Deardon, & Greaves, 2013), are more likely to be identified as having special educational needs (Gledhill, Ford, & Goodman, 2002; Martin, Foels, Clanton, & Moon, 2004), and as requiring speech‐language therapy services relative to older peers (Dockrell, Ricketts, & Lindsay, 2012). Younger children in a school year are also more likely to be diagnosed with behavioural problems (Goodman, 2003) including attention‐deficit/hyperactivity disorder (Morrow et al., 2012). The educational disadvantage experienced by younger children persists into secondary education and beyond (Cobley, McKenna, Baker, & Wattie, 2009).

An important question is what drives this age effect, as ameliorating it could substantially reduce the burden on public health services at a population level (Goodman, 2003). One possibility is that relative age represents a ‘season of birth’ effect, in which seasonal fluctuations in biological risk during pregnancy increase the risk of disadvantage at certain times of the year, perhaps due to mother's exposure to vitamin D or susceptibility to viruses (Hauschild, Mouridsen, & Nielsen, 2005). However, comparison of international findings provides strong evidence against this explanation as differences between youngest and oldest children in an academic year are observed across different countries with varying school entry cut‐off dates. For example, in Canada the cut‐off for school entry is 1st January, and autumn born children are the youngest at school entry. Here, autumn born children are more likely to be referred for psychiatric evaluation relative to summer born peers (Morrow et al., 2012), whereas the opposite pattern is evident in the United Kingdom (Goodman, Gledhill & Ford,^2003^).

Alternative explanations have focused on the age at which children start school or the age at which academic progress is assessed. In England the cut‐off date for school entry is 1 September; children typically start school in the academic year they become 5 years old. Thus, children born on 31st August start school at 4, while the oldest children in the class will be 5. Developmentally, 4‐year olds have more limited language and more immature emotional, social and behavioural skills relative to older peers. While there is no a priori reason to believe that younger children experience increased risk for clinically significant language difficulties, it is possible that these early developmental differences are compounded by classroom practices, such as an early focus on literacy and streaming by ability, which may lead to persistent inequalities.

In this regard, the relationship between language competence and behaviour may be informative. Recent changes to the National Curriculum in England have increased academic expectations in the first year of school. For instance, children are evaluated on their ability to listen attentively; follow instructions involving several ideas or actions; show awareness of listener needs; demonstrate confidence in speaking to their peer group; talk about their own and others feelings and behaviours and adjust their behaviour to the environmental context; read, write and understand simple written sentences; engage in verbal problem solving to complete doubling, halving and sharing maths problems; and to talk about size, weight, capacity, distance, time and money (Department for Education, 2013). If children start school with inadequate language to meet the social and academic demands of the classroom, behaviour problems may increase through frustration, peer difficulties and experience of failing at academic tasks. Consistent with this, Crawford, Dearden, and Greaves (2014) demonstrated that by age 8, older children in a year group held a significantly more positive view of their own academic competence relative to younger peers, even when actual academic attainment was equivalent. Thus, early school failure may have a negative impact on later attitudes to school and personal self‐esteem.

It is well established that language difficulties in the early school years also increase risk for later psychopathology (Petersen et al., 2013; Yew & O'Kearney, 2013). For instance, one‐third of children referred for tertiary psychiatric assessment are reported to have clinically significant, yet previously undetected language impairments (Cohen et al., 1998). In addition, children with language impairments are twice as likely as typically developing peers to show disorder levels of internalising problems, externalising problems and attention‐deficit/hyperactivity disorder (Yew & O'Kearney, 2013). However, most investigations concerning language and behaviour difficulties have focused on clinically referred cohorts; such samples are susceptible to Berkson's bias (a selection bias in which those with co‐occurring deficits are more likely to attract clinical attention) and may overestimate the extent to which language and behaviour difficulties are associated in the general population. Two large epidemiological studies reported that the relationship between early language difficulties and later psychopathology is mediated by comorbid reading disorders and associated school failure (Beitchman et al., 1996; Tomblin, Zhang, Buckwalter, & Catts, 2000). However, increased co‐occurrence of language and behaviour difficulties has also been observed at age 4 (Bretherton et al., 2014). This may indicate common underlying aetiology, and further suggests that some children starting school may not be able to regulate their behaviour and social interactions appropriately for the classroom.

There is considerable debate at policy level about how best to address relative age impacts. Crawford et al. (2014) advocated applying an age adjustment to educational achievement scores to overcome differences between the youngest and oldest children in a school year. However, adjusting scores may not be sufficient to reduce age‐related disadvantage, in part because it may not alter teacher perceptions of child competence or the child's own views of their academic abilities. The Department for Education in England is currently consulting about admissions policies that would enable a more flexible start date. This would allow the youngest children to start reception a year later than their oldest peers, a practice known internationally as ‘red‐shirting’ (Bedard & Dhuey, 2006). In theory, this should enable young children to develop language skills that are more commensurate with curriculum demands. However, the general consensus is that this practice is not effective for addressing relative age effects in academic attainment (Sharp, George, Sargent, O'Donnell, & Heron, 2009). It is also associated with socioeconomic status as only those families with the financial resources to fund an extra year of child care are able to hold their younger children back (Bedard & Dhuey, 2006). Finally, many experts and politicians have argued that raising the school starting age to 6 for all children would enable young children more time to develop the prerequisite skills (including language) needed for the early years curriculum (http://www.telegraph.co.uk/education/educationnews/10302249/Start-schooling-later-than-age-five-say-experts.html). In this regard it is worth noting that the United Kingdom has one of the lowest school starting ages in Europe; of 37 surveyed countries, 31 have start dates of 6‐years or later (Sharp et al., 2009).

In this study we seek to change the focus of the debate and ask whether the relative age effect reflects a mismatch between the developmental competencies of young children at school entry, and the developmental demands of the school curriculum. We employ the first UK‐based population study of risk of language impairment at school entry. We focus on language skills, as previous research has indicated that language skills at school entry are highly predictive of academic attainment at the end of formal education (Tomblin, 2008). Our first novel question asks whether relative age effects extend to teacher‐reported language abilities, after accounting for other factors associated with language deficit, including male sex, socioeconomic deprivation, exposure to English as an additional language (EAL) and behaviour problems. Our second question focuses on whether younger age is associated with co‐occurring language and behaviour difficulties, and whether those with co‐occurring deficits experience poorer academic progress. Our final question asks whether age accounts for unique variance in academic attainment once perceived language competence (and other demographic variables) are taken into account. The simultaneous measurement of language, behaviour and a nationally applied measure of academic attainment in a large population of children during their first year of formal education offers a unique opportunity to address these questions.

## Methods

### Study design

**Figure 1:**
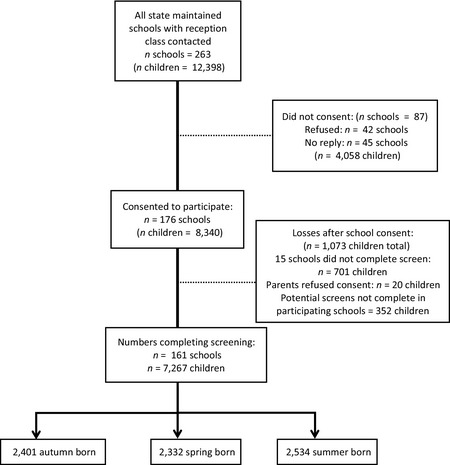
Recruitment flow chart. Numbers of potential participants calculated on basis of school census data of children enrolled in mainstream classrooms at beginning of 2011. Some children moved schools by summer 2012, contributing to incomplete screen numbers in participating schools

We conducted a population survey of children starting reception classes in state‐maintained primary schools. All state‐maintained primary schools in Surrey, England were invited to take part (*n* = 263) and data were obtained for 7,267 children who began a reception class in 2011 (61% of all eligible schools and 59% of all eligible children, Figure 1). There were no differences between schools taking part in the study and those that opt‐out with regard to the mean percentages of children receiving free school meals, (10.02% vs. 8.79%), t(261) = 1.38, *p* = .17; existing statements of special educational needs, (4.89% vs. 4.88%), t(261) = 0.19, *p* = .85; or speaking English as an additional language, (11.61% vs. 10.16%), t(232) = 1.05, *p* = .29. Notably, Surrey employs a single entry date for school admission, with virtually all children beginning school in the September of the academic year in which they turn 5. Thus, any differences in relative age are not confounded with length of time in school. However, it does mean that within our sample age at school entry, age at test, and ‘relative age’ are essentially the same.

The Research Ethics Committee at Royal Holloway, University of London approved the research protocol, which was developed in collaboration with Surrey County Council education authorities. Parents received information sheets indicating that anonymised teacher ratings of language, behaviour and educational attainment would be forwarded to the research team unless parents opted out. Twenty families opted out at this stage. The research team covered the cost of supply teaching for a day to enable teachers to complete the online screen for all children in the classroom.

### Participants

Children were aged between 4;9 (59 months) and 5;10 (70 months; mean = 64.16 months, *SD *= 3.55) at assessment, which occurred in the last term of the reception year (females = 3553, 49%; males = 3714, 51%). To allow comparison with previous investigations (Goodman *et al*., 2003), we divided the cohort into oldest (birthdays September to December), middle (birthdays January to April) and youngest cohorts (birthdays in May to August). Teachers reported that 782 (11%) of children were speakers of English as an Additional Language (EAL). Information was also obtained about existing clinical diagnoses (e.g. Down syndrome, autism spectrum disorder), and whether the child held a statement of special educational need, a legal document specifying educational support required for children with substantial developmental needs. As preexisting diagnoses and statements reflect significant concerns prior to school entry, these measures serve to demonstrate that any age‐related differences in our sample do not reflect a greater severity in one or more age groups prior to school entry (Table 1).

**Table 1:** Number (percentage) of children in each risk category by age group. The percentage of children in each risk category should be evenly distributed across age groups (i.e. 33%)

Measure	Oldest (*n* = 2401)	Middle(*n* = 2332)	Youngest (*n* = 2534)	Significance, *χ* ^2^
Male sex	1251 (33.7)	1188 (32.0)	1275 (34.3)	1.61, *p* = .45
English as additional language	260 (33.2)	261 (33.4)	261 (33.4)	1.02, *p* = .60
Low SES (IDACI rank)	244 (33.1)	235 (31.8)	259 (35.1)	0.03, *p* = .99
Existing medical/clinical diagnosis	49 (34.0)	49 (34.0)	46 (31.9)	0.58, *p* = .75
Statement of special educational need	37 (28.5)	42 (32.3)	51 (39.2)	1.56, *p* = .46
Language Difficulties (CCC‐S)^a^	150 (19.3)	256 (33.0)	371 (47.8)	91.25, *p* < .001
Behaviour Problems (SDQ‐Total difficulties)	201 (26.1)	262 (34.0)	308 (40.0)	20.03, *p* < .001
Not achieving ‘GLD’ (EYFSP)	582 (22.5)	818 (31.6)	1192 (46.0)	261.54, *p* < .001

a Percentages within each age group: oldest 6.25%, middle 10.98%, youngest 14.64%.

We obtained rank scores on the Income Deprivation Affecting Children Index (IDACI: http://www.education.gov.uk/cgi-bin/inyourarea/idaci.pl) from home postcodes provided by teachers. The IDACI score is a measure of neighbourhood deprivation reflecting the proportion of local children living with families who are in receipt of means tested benefits (McLennan et al., 2011), with a range in England of 1–32,482. While Surrey is more affluent than other English counties, our sample included a diverse population, with scores ranging from 731 (most deprived) to 32,474 (most affluent; mean = 21,592, *SD *= 7830). Children with scores in the bottom 10th percentile of our sample (9997 or less) were regarded as economically deprived. This is equivalent to the 31% most deprived areas in England, and is similar to the 30% cut used by the Department for Education (2014) as an indicator of poverty.

### Assessment measures

#### Children's Communication Checklist‐Short

The Children's Communication Checklist‐Short (CCC‐S) is a brief version of the CCC‐2 (Bishop, 2003). The full CCC‐2 is as effective as standardised assessment in identifying children with clinically significant language impairment (Bishop, Laws, Adams, & Norbury, 2006). The CCC‐S contains 13 items that best discriminated typically developing children from peers with language impairment in the validation study (Norbury, Nash, Baird, & Bishop, 2004), with high degrees of internal consistency (Cronbach's *α *= .95, this sample) and a significant correlation between CCC‐S and CCC‐2 total scores in the standardisation sample, Pearson's *r*(515) = .88. Each item provides an example of language behaviour in everyday contexts and covers speech, vocabulary, grammar and discourse. Teachers rated the frequency with which these behaviours occur on a 4‐point scale, with higher scores reflecting greater communication difficulites. CCC‐S scores within our sample spanned the full range of possible scores (0–39; mean = 9.34, *SD *= 9.09). Children scoring 1.25 *SD* above the mean (90th centile; raw score of 22 or greater) were deemed to have significant concern about language; this cut‐off has been associated with long‐term risk of academic and social disadvantage (Reilly et al., 2014).

#### Strengths and Difficulties Questionnaire

The Strengths and Difficulties Questionnaire (SDQ) is a well‐validated screening measure of children's social, emotional and behavioural functioning, with good reliability, construct validity and capacity to identify children who have clinically significant behaviour problems (Goodman, 1997; Stone, Otten, Engels, Vermulst, & Janssens, 2010). The SDQ is comprised of 25 items across five subscales: emotional symptoms, conduct problems, hyperactivity, peer problems and prosocial behaviour. Teachers rated child behaviour on a 3‐point scale, with higher scores reflecting increased behaviour difficulties. A Total Difficulties score was derived by summing the first four subscales (maximum score 40, range in our sample 0–35, mean = 5.48, *SD *= 5.21) and had excellent levels of internal consistency (Cronbach's *α *= .90, this sample). For comparison with the CCC‐S, we identified a categorical cut‐off for problem behaviour at the 90th centile (raw scores of 13 or greater).

#### Early Years Foundation Stage Profile

The Early Years Foundation Stage Profile (EYFSP) is a statutory assessment of academic progress in English primary schools administered at the end of the reception year (Department for Education, 2013). The EYFSP includes 17 attainment targets that are rated on a 3‐point scale as ‘emerging’ (1 point), ‘expected’ (2 points), or ‘exceeding’ (3 points). Scores within our sample spanned the entire range from 17–51 (mean = 35.32, *SD *= 7.81; Cronbach's *α *= .96, this sample), with lower scores reflecting educational concern. In addition, a Government defined index of ‘Good Level of Development (GLD)’ requires ‘expected’ or ‘exceeded’ targets on 12 key curriculum targets including personal, social and emotional development; physical development; language and communication; mathematics and literacy (Cotzias & Whitehorn, 2013).

### Missing data

Household postcodes were not available for 205/7267 children and were replaced with the postcode for the child's school. One child was missing both SDQ and EYFSP scores and six were missing EYFSP due to teachers exiting the online screen before completion. The screen required a response to each individual item before teachers could progress to the next item, thus there were no further missing data.

### Statistical analysis

Statistical analyses were implemented in Stata 12. Our first question examined the relationship between age group, language competence and other risk variables using *χ* ^2^ and logistic regression for categorical outcome (language deficit, i.e. CCC‐S scores of 22 or greater, vs. adequate language). If age was not associated with language, we would expect language deficits to be evenly distributed across the age groups (i.e. 33% of the oldest, middle or youngest cohorts). We used the middle age group as the reference group as a more conservative estimate of risk. It also enabled us to determine whether older children were significantly advantaged in language ability, as well as investigating disadvantage for the youngest group. All variables were entered simultaneously in the regression analysis; these included age group, male sex, lower socioeconomic status, EAL and behaviour problems. Our second question considered the relationships between language and behaviour. We report the percentages of children achieving a good level of development on the EYFSP (Cotzias & Whitehorn, 2013) according to language/behaviour status (no risk, behaviour difficulties only, language difficulties only, co‐occurring language and behaviour difficulties). Our final question investigated these relationships using continuous variables. We conducted a linear regression with EYFSP total score as the outcome variable, to estimate the relative contributions of age, language competence and behavioural skills (as well as other demographic variables) to academic attainment.

## Results

Age group was not associated with any sociodemographic variable, nor was it significantly associated with existing clinical diagnosis or current statement of special educational need (Table 1). This indicates that the youngest children were not significantly disadvantaged prior to school entry. However, the youngest children in the class were more likely to have significant behaviour problems reported and were the least likely to achieve a Good Level of Development on the EYFSP.

**Figure 2:**
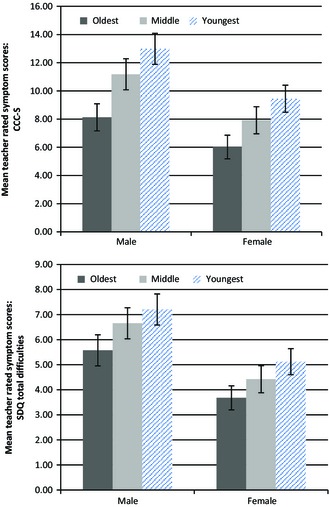
Associations between age of children and mean symptom score on the CCC‐S (top) and SDQ‐Total Difficulties score (bottom) by age group and sex. Error bars represent 95% confidence intervals

The results also show for the first time a significant association between teacher ratings of language difficulty and age group. Of those with teacher‐rated language difficulties, 32.9% were in the middle age group, exactly the proportion expected by chance. In contrast, only 19.3% were in the oldest cohort, while 47.7% of all children with reported language difficulties were in the youngest cohort; more than twice as in the oldest group. Although males generally obtained higher (i.e. worse) scores compared with females on the CCC‐S and the SDQ, the effect of age group is apparent in both sexes (Figure 2).

Binary logistic regression demonstrated that age group remained a significant predictor of language status after adjustment for the other significant risk factors (Table 2). The oldest children in the cohort were at significantly reduced risk of teacher‐rated language difficulties relative to the reference group; adjusted odds ratio: 0.55, 95% CI [0.44, 0.69]. In contrast, the youngest children were at significantly greater risk relative to peers; adjusted odds ratio: 1.46, 95% CI [1.21, 1.76]. The overall model provided adequate fit to the data, Hosmer–Lemeshow *χ* ^2^ (7) = 10.55, *p* = .16, and explained a significant, though modest, amount of variance (McFadden's pseudo *R* square = .18).

**Table 2:** Binary logistic regression predicting teacher ratings of language difficulties in 90th centile and above. The middle age group is used as the reference category for calculating effect of age group. All variables are significant individual predictors at *p* < .001

	*B*	*SE*	*Z*	Odds ratio	95% CI	
Oldest	−0.60	.12	−5.24	0.55	0.44 0.69
Youngest	0.38	.10	4.00	1.46	1.21 1.76
Male sex	0.54	.09	6.17	1.72	1.44 2.03
EAL	1.39	.10	13.39	4.02	3.28 4.93
Low SES	0.50	.12	4.31	1.65	1.31 2.07
Behaviour problems	2.16	.09	23.20	8.70	7.25 10.45
Constant	−3.17	.10	32.05	0.04		

**Figure 3:**
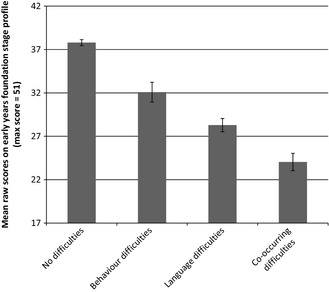
Effects of language deficit and behaviour problems on raw scores of the EYFSP (minimum score 17, maximum score 51). Bars indicate 95% confidence intervals

With respect to language and behaviour, reported behaviour problems were highly associated with language deficits; adjusted odds ratio: 8.70, 95% CI [7.25–10.45]. Children with CCC‐S scores above 90th percentile and SDQ‐Total Difficulties scores above 90th percentile were deemed to have co‐occurring deficits. Younger age was also associated with co‐occurring language and behaviour deficits (youngest: *n* = 135, middle: *n* = 108 and oldest: *n* = 72); almost twice as many of the youngest children had both language difficulties and behaviour problems relative to older children, reflecting the increased incidence of language difficulties in this group, *χ* ^2^(6) = 106.90, *p* < .0001. Figure 3 illustrates the impact of language and behaviour problems on academic attainment. Only 4.8% of children with language only difficulties and 1.3% of those with co‐occurring language and behaviour deficits achieved a Good Level of Development on the EYFSP, relative to 67.1% of those with no risk indicators and 20.7% of those with behaviour difficulties only. However, it is worth noting that across the population, only 57% of children achieved a Good Level of Development on the EYFS Profile, which is comparable to the 52% of children achieving a Good Level of Development in an audit of the new EYFSP by the UK government (Cotzias & Whitehorn, 2013).

Finally, we conducted a linear regression to investigate the extent to which age predicts unique variance in academic attainment after accounting for demographic variables, language and behaviour. Table 3 shows that together these factors accounted for 52% of the variance in teacher‐rated educational attainment at the end of the reception year, and that each factor accounts for significant unique variance. Although this further illustrates the impact of age at school entry on early academic attainment, the size of this effect is small, accounting for 1% of the variance in EYFSP scores (semipartial *r *=* *.11). In comparison, language skills accounted for the largest percentage (19%) of unique variance in teacher‐rated scholastic achievement (semipartial *r* = −.43).

**Table 3:** Linear regression predicting EYFSP scores from demographic variables, teacher ratings of language competence and teacher ratings of behavioural difficulties

	*t*	Beta	Semipartial *r*
Age	13.26**	0.11	0.11
Sex	−5.33**	−0.04	−0.04
SES	8.09**	0.07	0.07
EAL	2.52*	0.02	0.02
CCCS total	−53.23**	−0.54	−0.43
SDQ‐Total Difficulties	−20.97**	−0.21	−0.17
*R* ^2 ^= .52, *p* < .001			

**p < .001; *p < .05.

## Discussion

Consistent with previous research (Department for Education, 2014; Goodman *et al*., 2003), the youngest children were at increased risk of behaviour problems and poor academic attainment, even in their first year of formal schooling. A novel finding from our population study is that in the first year of school, the youngest children were perceived by teachers to have lower levels of language competence and there were more instances of reported co‐occurring language and behaviour problems. In addition, only 1.3% of those with language and behaviour problems obtained a good level of academic development at the end of their first year of school.

Our findings suggest that the classroom experience may disadvantage the youngest children. An important question is why? Our data argue against a season of birth explanation as medical diagnoses and statements of special educational need prior to school entry did not differ significantly across the age groups.

Others have argued that age at test explains these effects (Crawford et al., 2014). It is perhaps not surprising that teachers rated younger children as less competent relative to peers who are 12 months older. Recently, there have been calls to adjust educational assessments for age (Crawford et al., 2013, 2014). This may not ameliorate the relative age effect however, because younger children still may not have sufficient language skills to meet the daily social and academic demands of the classroom and this in turn may affect their behaviour, social development and attitude to learning. It is also possible that immature language at school entry is a marker for other cognitive and behavioural concerns that further challenge classroom learning. Longitudinal studies are needed to elucidate these causal pathways.

Teachers are charged with ensuring that all children in the class meet a prespecified list of learning targets, whatever their birthdate. Our results question whether many of the youngest children in the classroom have the language skills to meet the demands of the curriculum, to integrate socially with older peers and to regulate their own emotions and behaviours. In this regard, it is important to note that relative age effects were also observed in the UK Government's audit of the new EYFSP (Cotzias & Whitehorn, 2013). Of potentially greater concern, only 52% of children nationally achieve a Good Level of Development on the EYFSP, similar to our estimate of 57% in a relatively affluent county. It would appear that curriculum targets are out of line with developmental expectations at this age. However, in our sample it is not possible to distinguish between the effects of relative age, age at school entry and age at test, as all children were assessed in the final school term and thus the youngest in the class were also the youngest when assessed.

### Clinical implications

Our findings do not provide clear guidance about the optimal age at which a child should start school, or whether deferring school entry for a summer‐born child will benefit that individual. The majority of European countries begin compulsory education at the age of 6 or 7, though many provide state‐funded nursery provision at an earlier age. Previous research has demonstrated that deferring school entry (‘red‐shirting’) is associated with socioeconomic advantage; more educated families and those with the financial resources to fund an extra year of child care are more likely to defer school entry (Bedard & Dhuey, 2006). Thus, if this practice were widespread, it could further serve to disadvantage vulnerable children, who by virtue of their impoverished social circumstances are already at increased risk of language impairment, behaviour difficulties and slow academic progress.

Organising class groups by ability appears to compound the effects of relative age (Bedard & Dhuey, 2006), by reinforcing teacher perceptions of younger children as less capable or compliant, even though their language and behaviour may be within the wide range expected for age. Organising reception classes by age group might be beneficial in highlighting to teachers which children are the youngest and allowing them to adjust their expectations accordingly. Simpler interventions such as calling the class register by birthdate may also achieve the same effect (Goodman, *et al*., 2003). Importantly, these measures may also serve to highlight older children with developmental deficits. Our findings demonstrate that older children were significantly less likely to be identified by teachers despite similar proportions of clinical diagnosis and educational need prior to school entry.

We offer a new suggestion that relative age effects might be tempered by ensuring that curriculum targets are more closely matched to the developmental competencies of children at school entry. Specifically, our data indicate the need to adapt the early years curriculum to focus on developing children's oral language skills, social competencies and behaviour control. A focus on oral language in reception might also serve to underpin later literacy instruction. Improving oral language skills can result in improvements in text reading and text comprehension (Fricke, Bowyer‐Crane, Haley, Hulme, & Snowling, 2013). Delaying the start of literacy instruction until age 7 does not impede long‐term reading achievement, may increase positive attitudes to literacy instruction and improve reading comprehension (Suggate, Schaughency, & Reese, 2013). Furthermore, Scandinavian countries do not begin literacy instruction until ages 6–7, enjoy high standards of literacy and do not show evidence of relative age effects in international assessment (Bedard & Dhuey, 2006). Thus, being the youngest at school entry may not be problematic if the curriculum targets are more consistent with developmental capacities.

### Strengths and limitations

A major strength of our study is the large population cohort, all of whom were in the same year group and had been attending school for the same amount of time. Unlike previous studies of relative age, we were able to link our measures of language and behaviour to a universally applied measure of academic achievement, allowing us to assess the functional impact of low scores on our teacher report questionnaires. Although the CCC‐S and SDQ are likely to provide an accurate picture of developmental concern, our study is limited by the lack of direct measurement of language and behaviour. Reliance on indirect measurement strategies introduces concern about common method variance. In particular, the relationship between language and behaviour difficulties might be inflated in our study by the tendency of teachers to notice more readily those children who are disruptive in the classroom. Thus, multiple informants and direct assessment of child language and behaviour will further elucidate their relationships and the importance of relative age in cementing those relationships. Nevertheless, as teacher perception of language competence and behavioural compliance is highly influential in classroom practices that might exacerbate relative age effects, our findings have important ecological validity.

## Conclusion

This study provides compelling evidence that younger children in reception classes are perceived to have lower levels of language competence, more behaviour problems and more limited academic progress than older peers. We suggest that these challenges reflect a mismatch between developmental competence and academic expectations. Different strategies to address this concern could be evaluated using randomised controlled trials. While the unique contribution of age is small, strategies that effectively attenuate the relative age effect could reap substantial savings to clinical and education budgets at a population level. Approximately 730,000 children are born in England each year, and our data suggest a 50% increase in the number of younger children identified as having possible language deficits at the end of reception. Thus, an extra 36,500 children could be identified as having poor language, behaviour problems and educational difficulties in their first year of school, simply because of their younger age. Reducing the level of difficulty experienced by the youngest children in the class could therefore enable scarce clinical resources to be targeted more effectively.

**Key points**

- Younger children in a school year are at higher risk of educational adversity and psychiatric disorder.
- Clinically significant language impairment also confers broad risk for emotional and behavioural disorder and scholastic underachievement.
- In this first UK population study of language at school entry, younger age is associated with teacher perceptions of poorer language competence and co‐occurring language and behavioural problems.
- Young age is also associated with poorer academic progress in the first year of school, though language ability is the best indicator of scholastic achievement.
- Fewer than 5% of children with language and behavioural deficits achieve good academic progress in their first year of school.
- Younger children at school entry may not have sufficient language and behaviour skills to meet the academic and social demands of the education system, creating increased need for specialist clinical resources.
- At a population level, reducing academic practices that exacerbate the age effect and enhancing oral language proficiency in the early years should reduce referrals to specialist clinical services.
